# Lateralization of Bilateral Temporal Lobe Epilepsy After Vagus Nerve Stimulation Surgery

**DOI:** 10.7759/cureus.111907

**Published:** 2026-07-01

**Authors:** Hsiang-Yu Wu, Po-Wei Chen, Chien Chen, Hsin-Hung Chen

**Affiliations:** 1 Department of Neurosurgery, Neurological Institute, Taipei Veterans General Hospital, Taipei, TWN; 2 Department of Neurology, Neurological Institute, Taipei Veterans General Hospital, Taipei, TWN

**Keywords:** anterior temporal lobectomy, bitemporal epilepsy, drug-resistant epilepsy, lateralization, vagus nerve stimulation

## Abstract

Drug-resistant bilateral temporal lobe epilepsy is associated with poor outcomes following resective surgery because of the lack of a clearly lateralized focus. Vagus nerve stimulation (VNS) provides symptomatic benefit, but its potential role in facilitating surgical candidacy remains underrecognized. We describe a 32-year-old woman who developed drug-resistant bilateral temporal lobe epilepsy after herpes simplex virus encephalitis at age 13. Seizures included dialeptic, hypermotor, and generalized tonic-clonic events without consistent lateralizing features. Scalp electroencephalography showed bilateral independent temporal discharges, and magnetic resonance imaging revealed bilateral mesial temporal atrophy. At age 19, she underwent VNS implantation. Subsequent electroencephalography demonstrated progressive lateralization of interictal and ictal discharges to the right temporal lobe. At age 29, she underwent right anterior temporal lobectomy (ATL), resulting in Engel Class I seizure freedom. This case illustrates the potential of VNS not only as a palliative therapy but also as an inducer of lateralization of discharges, thereby enabling patients to undergo curative resective surgery.

## Introduction

Unilateral temporal lobe epilepsy is the most common focal epilepsy syndrome in adults and is often amenable to surgical intervention. For patients with drug-resistant (seizure persistence despite at least two antiseizure medications) unilateral temporal lobe epilepsy, anterior temporal lobectomy (ATL) yields favorable outcomes, with reported Engel Class I seizure freedom rates (complete freedom from disabling seizures) ranging from 58% to 89% [1-3]. In contrast, bilateral temporal lobe epilepsy presents a greater therapeutic challenge. These patients frequently exhibit non-lateralizing semiology and bilateral epileptiform discharges, making them less suitable candidates for resective surgery. A systematic review reported that only 23% of bilateral temporal lobe epilepsy patients achieved Engel Class I outcomes following surgical treatment [4]. For individuals with drug-resistant bilateral temporal lobe epilepsy who are not optimal candidates for resection, neuromodulation therapies (treatments altering nerve activity through electrical stimulation), such as vagus nerve stimulation (VNS), have emerged as viable alternatives [5].

## Case presentation

The patient is a 32-year-old woman with a history of herpes simplex virus encephalitis at the age of 13, followed by the onset of epilepsy. Her seizure semiology included dialeptic episodes, vocalization, hypermotor activity, and occasional generalized tonic-clonic seizures, occurring 5-10 times per month. Reported triggers included psychological stress and sleep deprivation. She was treated with oxcarbazepine 600 mg every 12 hours, lamotrigine 250 mg every 12 hours, and clobazam 20 mg at bedtime.

She presented to our epilepsy clinic at the age of 15. Brain magnetic resonance imaging demonstrated poorly defined gray-white matter differentiation in the bilateral hippocampal body, suggestive of focal mesial temporal sclerosis (Figure 1). Interictal scalp electroencephalography revealed bilateral temporal epileptiform discharges (Figures 2-3). Semiology captured on video electroencephalography demonstrated behavioral arrest without clear lateralizing features. Ictal electroencephalography showed ictal onset from the left temporal region (Figure 4). Neither positron emission tomography-computed tomography nor stereotactic electroencephalography was performed at that time. Based on the clinical presentation, imaging, and electroencephalography findings, a diagnosis of drug-resistant bilateral temporal lobe epilepsy was established.

**Figure 1:**
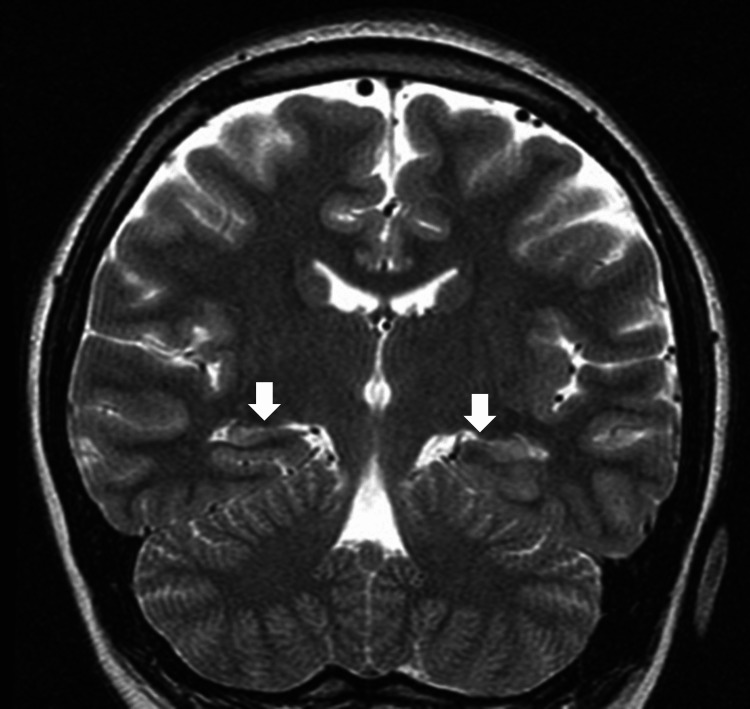
Brain magnetic resonance imaging before VNS surgery. The T2-weighted image showed bilateral mesial temporal atrophy, suggestive of mesial temporal sclerosis. VNS: vagus nerve stimulation

**Figure 2:**
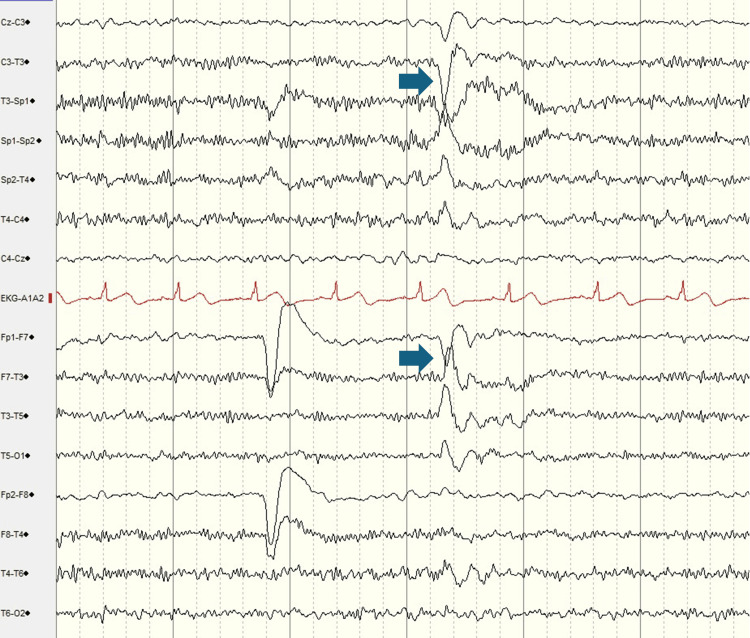
Interictal electroencephalography (1) before VNS surgery. ST montage showing epileptiform discharges over the left temporal region. VNS: vagus nerve stimulation

**Figure 3:**
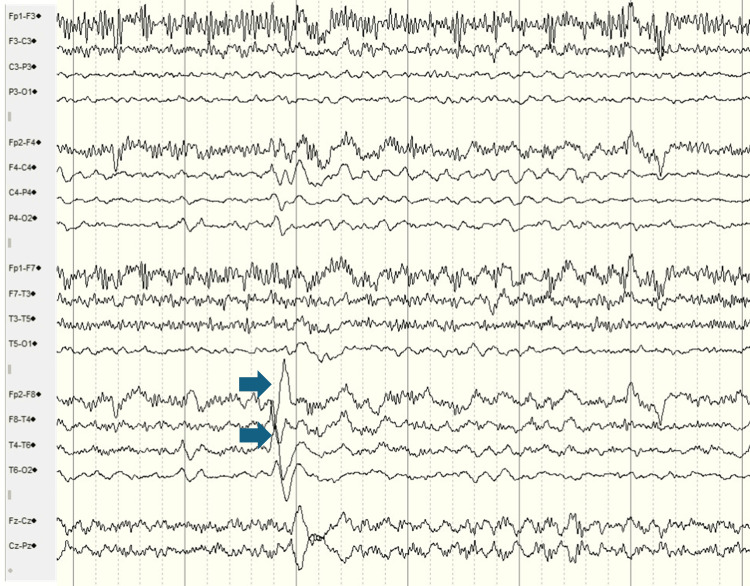
Interictal electroencephalography (2) before VNS surgery. Bipolar montage showing epileptiform discharges over the right anterior temporal region. VNS: vagus nerve stimulation

**Figure 4:**
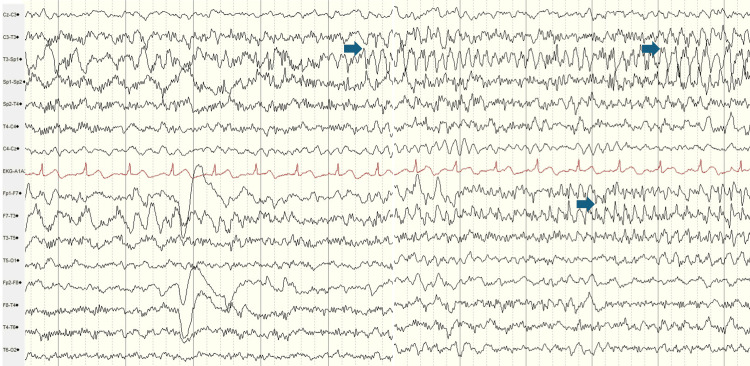
Ictal electroencephalography before VNS surgery. ST montage showing theta activity in the left temporal region. VNS: vagus nerve stimulation

VNS surgery was performed on the left side when the patient was 19 years old. The stimulation parameters were set at 2 mA, 25 Hz, 500 µsec pulse width, with a cycle of 30 seconds on and five minutes off. Following VNS implantation, seizure frequency decreased to one to three episodes per month, and generalized tonic-clonic seizures were no longer observed.

Positron emission tomography-computed tomography performed at ages 22 and 28 demonstrated hypometabolism in the bilateral temporal lobes, more pronounced on the right (Figure 5). However, no pre-VNS image was available for comparison.

**Figure 5:**
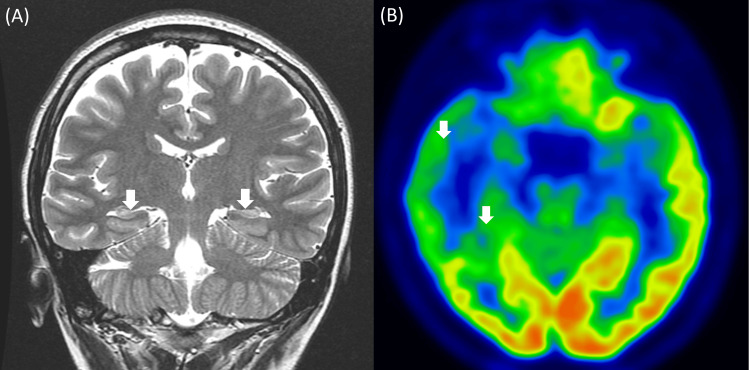
Brain imaging before ATL surgery. (A) T2-weighted magnetic resonance image showing bilateral mesial temporal atrophy, suggestive of mesial temporal sclerosis. (B) Positron emission tomography image showing decreased fluorodeoxyglucose uptake in the right temporal lobe. ATL: anterior temporal lobectomy

Follow-up interictal electroencephalography revealed epileptiform discharges exclusively in the right temporal region (Figure 6). Seizure semiology during VNS activation included hypermotor behavior and vocalization, with ictal electroencephalography showing right temporal onset (Figure 7). Interestingly, seizure semiology during VNS deactivation differed; in addition to hypermotor behavior and vocalization, head turning to the left and generalized tonic-clonic seizures were observed. Corresponding ictal electroencephalography during VNS-off periods also showed right temporal epileptiform discharges.

**Figure 6:**
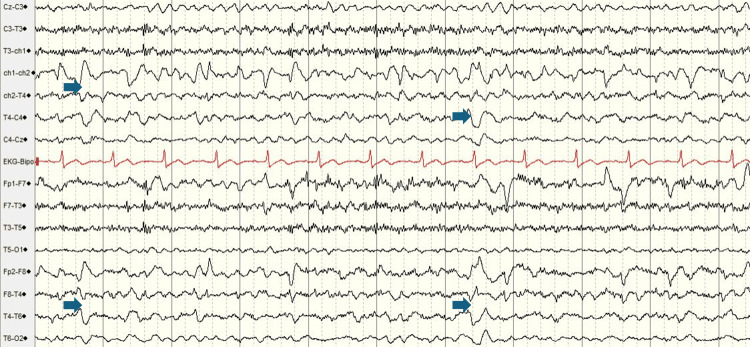
Interictal electroencephalography before ATL surgery. ST montage showing epileptiform discharges over the right temporal region. ATL: anterior temporal lobectomy

**Figure 7:**
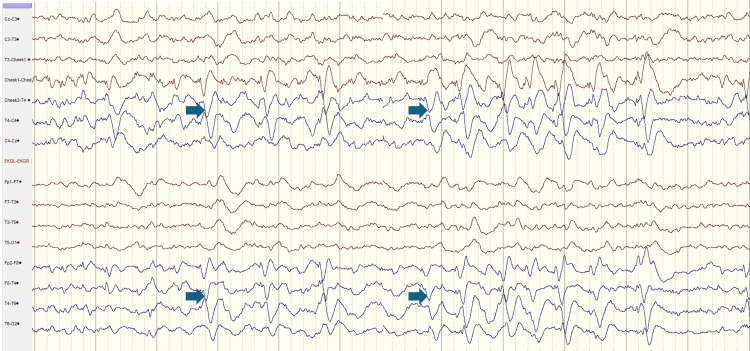
Ictal electroencephalography before ATL surgery. ST montage showing epileptiform discharges over the right temporal region. ATL: anterior temporal lobectomy

Based on the evolving semiology, interictal and ictal electroencephalography findings, and positron emission tomography-computed tomography imaging, it was hypothesized that VNS induced functional lateralization of epileptiform activity to the right temporal lobe. The patient subsequently underwent right ATL at age 29, with a resection extent of 5 cm from the temporal tip, including the amygdala and hippocampus. Histopathological analysis confirmed mesial temporal sclerosis. Postoperatively, the patient achieved Engel Class I seizure outcome, and follow-up interictal electroencephalography at one year showed no epileptiform discharges.

Interestingly, the VNS battery was depleted at age 33, after which the patient began experiencing occasional dialeptic seizures. Ictal electroencephalography during these episodes demonstrated 10-20 seconds of semirhythmic theta and delta activity over the left temporal region, evolving to bilateral hemispheric involvement. This finding further supports the hypothesis that the suppression of left hemispheric epileptiform activity prior to ATL was mediated by active VNS. The recurrence of left-sided discharges following battery depletion suggests a reversible lateralizing effect of VNS. Table 1 presents the timeline of the patient's clinical course by age.

**Table 1:** Timeline of the patient's clinical course by age.

Timeline	
Age 13: Onset of Epilepsy	
Age 13	VNS Status: N/A (Pre-implantation)
	Seizure Phenotype: Dialeptic episodes, vocalization, hypermotor activity, and occasional generalized tonic-clonic seizures (5-10 times/month).
Age 15	EEG Finding: Bilateral temporal epileptiform discharges.
	Brain MRI: Suggestive of bilateral focal mesial temporal sclerosis.
	Diagnosis: Drug-resistant bilateral temporal lobe epilepsy.
Age 19: Left VNS Implantation	
Age 19	VNS Status: On (left-side, 2 mA, 25 Hz, 500 µsec, 30s on with 5min off)
	Seizure Phenotype: (1-3 times/month); GTCS no longer observed.
	EEG Finding: Right temporal epileptiform onset.
Age 22/28	PET-CT: Bilateral temporal lobe hypometabolism, pronounced on the right.
	Hypothesis: VNS induced functional lateralization of epileptiform activity to the right temporal lobe.
Age 29: Right Anterior Temporal Lobectomy	
Age 29	VNS Status: On
	Pathology: confirmed mesial temporal sclerosis.
	Outcome: Achieved Engel Class I seizure freedom. Follow-up interictal EEG at 1 year showed no epileptiform discharges.
Age 33: VNS Battery Depletion	
Age 33	VNS Status: Depleted (Off)
	Seizure Phenotype: Recurrence of dialeptic seizures.
	EEG Finding: 10-20 seconds of semirhythmic theta and delta activity over the left temporal region, evolving to bilateral hemispheric involvement.

## Discussion

In patients with drug-resistant epilepsy who are not candidates for resective surgery or whose seizures persist despite surgical intervention, neuromodulation therapies such as VNS are recommended [5]. The VNS system consists of a lead wrapped around the left cervical vagus nerve within the carotid sheath, connected to a pulse generator implanted subcutaneously in the chest, typically below the left clavicle. Stimulation of the right vagus nerve is generally avoided due to the higher risk of bradyarrhythmias, as the right vagus nerve exerts more direct influence on sinoatrial node function. This precaution is primarily based on animal studies [6-8].

Several prognostic factors associated with favorable outcomes following VNS therapy have been identified, reflecting a complex relationship between seizure networks and stimulation response. In a large retrospective analysis of 5,554 patients from the Vagus Nerve Stimulation Therapy Patient Outcome Registry, clinical predictors for seizure freedom included an age of epilepsy onset >12 years (odds ratio: 1.89; 95% confidence interval: 1.38-2.58) and a predominantly generalized seizure type (odds ratio: 1.36; 95% confidence interval: 1.01-1.82), while a better overall response (≥50% seizure reduction) was predicted by nonlesional epilepsy (odds ratio: 1.38; 95% confidence interval: 1.06-1.81) [9].

When evaluating focal network profiles, however, smaller studies present conflicting evidence regarding the predictive result of discharge lateralization. On the one hand, data support the premise that lateralized epilepsy type (unilateral) yields better VNS outcomes; Janszky et al. analyzed 47 patients with left-sided VNS implantation and found that absence of bilateral interictal epileptiform discharges was associated with seizure-free outcome (p=0.01, odds ratio: 29.2, 95% confidence interval: 2.4-353) [10], which means that left-sided VNS leads to seizure-free status only in patients with lateralized epileptiform discharge. Interestingly, five of the six patients who had become seizure-free from left-sided VNS had discharges localized to the left hemisphere [10,11]. Similarly, in a retrospective study involving 144 patients receiving VNS, Ghaemi et al. reported that unilateral interictal epileptiform discharges were predictive of seizure freedom (p = 0.014, odds ratio: 0.112, 95% confidence interval: 0.019-0.642) [12].

On the other hand, the therapeutic result of VNS in managing widespread, bilateral epilepsy remains substantial. In a small cohort of 17 patients, Dede et al. conversely observed that bilateral ictal and interictal electroencephalography was associated with better outcomes [13]. This efficacy in bilateral syndromes is further quantified by Alshahrani et al., who, in another study, reported that 12 of 17 patients (70.5%) with bilateral temporal lobe epilepsy achieved ≥50% seizure reduction after VNS, and 10 patients (58.8%) achieved ≥75% reduction [14]. Taken together, these variable responses underscore that, while unilateral focus patterns historically favor optimal suppression, bilateral networks remain highly responsive to neuromodulatory intervention, highlighting a gap in our understanding of how VNS progressively modulates bi-hemispheric excitability.

In summary, VNS appears to be beneficial in patients with bilateral temporal lobe epilepsy; however, evidence regarding its potential lateralizing effect remains limited and inconsistent. To our knowledge, no prior case has been reported in which a patient became eligible for resective surgery following VNS therapy. In our case, bilateral temporal epilepsy functionally lateralized to the right hemisphere after VNS implantation. Two possible mechanisms may explain this observation. First, the disappearance of left-sided epileptiform discharges may reflect a true lateralizing effect of VNS, as suggested by Janszky et al. [10]. Alternatively, the patient may have had right temporal lobe epilepsy with rapid contralateral synchronization, resulting in apparent bilateral onset. VNS may have suppressed this rapid propagation, thereby unmasking the true epileptogenic focus.

Several limitations of this case report should be acknowledged. First, the interpretation of the patient's metabolic evolution is slightly hampered by the lack of a baseline, pre-implantation positron emission tomography-computed tomography scan. Without this initial neuroimaging, a true "before-and-after" quantitative comparison of bilateral temporal lobe metabolism was not possible, forcing us to rely on post-VNS imaging at later follow-ups. Second, although the evolving seizure semiology provided crucial clinical clues during VNS activation and deactivation, semiology alone is inherently insufficient to definitively differentiate true bilateral temporal lobe epilepsy from unilateral temporal epilepsy with rapid contralateral propagation. While the recurrence of left-sided ictal discharges following VNS battery depletion strongly suggests a reversible neuromodulatory effect, the inclusion of pre-implantation stereotactic electroencephalography would have provided definitive electrophysiological proof of the baseline networks.

## Conclusions

We report a unique case of bilateral temporal lobe epilepsy in which the patient became eligible for anterior temporal lobectomy following palliative VNS. Initially presenting with bilateral temporal discharges, the patient's electroclinical profile shifted entirely to the right temporal lobe following left-sided VNS implantation. This clear lateralization provided a clinical window of opportunity that allowed for a successful right anterior temporal lobectomy, yielding an Engel Class I surgical outcome. This outcome suggests that VNS may facilitate functional lateralization or unmask the primary epileptogenic focus in patients initially diagnosed with bilateral involvement. Further studies are warranted to elucidate the mechanisms underlying this phenomenon.
